# Supplementary material for: High antibody titres induced by protein subunit vaccines using Mycobacterium ulcerans antigens Hsp18 and MUL_3720 with a TLR-2 agonist fail to protect against Buruli ulcer in mice
Source: PeerJ. 2020 Aug 7;8:e9659. doi: 10.7717/peerj.9659 (PMC7416718; doi:10.7717/peerj.9659)
Supplement: Supplemental Information 4 [file peerj-08-9659-s004.docx]

Supplementary Table 4. *M. ulcerans* challenge survival outcomes in vaccinated BALB/C mice.

| **Vaccination group** | **# mice with ulcers at day 63 (n=7)** | | **# mice with ulcers at day 68 (n=7)** | | **# mice with ulcers at day 75 (n=7)** | | |
| --- | --- | --- | --- | --- | --- | --- | --- |
| MUL_3720 + R_4_Pam_2_Cys | | 0 | | 3 | | 7 |  |
| Hsp18 + R_4_Pam_2_Cys | | 0 | | 4 | | 7 |  |
| Mul_3720 alone | | 0 | | 6 | | 7 |  |
| R_4_Pam_2_Cys alone  (without MUL_3720) | | 0 | | 7 | | - |  |
| Hsp18 alone | | 0 | | 7 | | - |  |
| R_4_Pam_2_Cys alone  (without Hsp18) | | 0 | | 2 | | 7 |  |
| BCG | | 0 | | 5 | | 7 |  |
